# Youth insights on menstrual health and hygiene in Burkina Faso; a comparison of written versus spoken qualitative text

**DOI:** 10.1371/journal.pgph.0006804

**Published:** 2026-09-11

**Authors:** Temidayo Akinreni, Teresa Buitrago-García, N. Hélène Sawadogo, Aurélia Souares, Mark Donald C Reñosa, Kerry Scott, Ali Sié, Sarah Langlotz, Till Bärnighausen, Shannon A McMahon

**Affiliations:** 1 Heidelberg Institute of Global Health, Ruprecht-Karls Universität Heidelberg, Heidelberg, Germany; 2 Madrilenian Health Service, Madrid, Spain; 3 Nouna Health Research Centre, Namory Kéita, Nouna, Burkina Faso; 4 Department of Epidemiology and Biostatistics, Department of Health, Research Institute for Tropical Medicine, Muntinlupa, Philippines; 5 School of Global Health, Faculty of Health, York University, Toronto, Canada; 6 Chair of Development Economics, Georg-August-Universität Göttingen, Göttingen, Germany; Rollins School of Public Health: Emory University School of Public Health, UNITED STATES OF AMERICA

## Abstract

Understanding girls’ lived experiences with menstruation is essential yet can be challenging given the taboo nature of the topic. While diaries have not previously been employed as a data collection method among girls in this setting, the use of diaries could generate unique insights due to their private, reflective, and participant-led nature. Our study aimed to explore the menstrual health and hygiene experiences of schoolgirls in Burkina Faso, comparing diaries and in-person interviews, and to capture girls’ thoughts on the process and value (or lack thereof) of diary writing. This study involved 30 girls aged 10–19 years to understand their experiences of menstruation. Data were analyzed using inductive and deductive approaches within a systematic thematic analysis framework, supported by NVivo 12 pro. Common social meanings attached to menarche include “becoming a woman” and “staying away from boys”. Bullying, stigma, and inadequate water, sanitation, and hygiene facilities were identified as major challenges in schools. For some, the diary provided a safe space to express their thoughts and share their menstruation stories. However, challenges with diary writing included its additional workload, limitations linked to language proficiency and writing ability, and perceptions of the diaries as being repetitive in terms of questions. Diaries and IDIs generated similar menstruation-related themes, indicating that they are equally effective in capturing experiences on this topic among this respondent group in this setting. Our study reinforces the need to support schoolgirls in achieving menstrual health and wellbeing. Efforts should prioritize promoting education across communities to mitigate the impact of cultural and religious restrictions. In contexts where participants have limited writing and language proficiency, future studies may consider alternative methods that nevertheless facilitate privacy and are respondent-driven, such as audio diaries. Further research could benefit from adopting either method of data collection to fit the contextual needs of participants.

## Background

Despite ongoing efforts and interventions channeled to improve menstrual health and hygiene, adolescent girls, especially in resource-limited settings, continue to face substantial challenges [1]. Research indicates that more than half of menstruating women and girls lack essential resources such as menstrual products (disposable pads, reusable products, etc) and access to proper water, sanitation, and hygiene (WASH) facilities [2]. This gap has far-reaching health implications, contributing to menstrual and reproductive health issues and heightening the risk of sexual health complications [1,3–5].

Similar to girls in many Sub-Saharan African countries, girls in Burkina Faso face major challenges in terms of addressing their menstrual health. A 2017 national survey revealed that only 26% of women had access to all necessary resources for effective menstrual management [6]. Studies have also found that approximately two-thirds of women and girls rely on cloth for menstrual health and hygiene [6,7]. While cloth pads are not inherently harmful, improper washing and drying practices, or the use of unclean water, can lead to infections, as found in other studies [8].

Previous research from several Sub-Saharan African countries indicates that some girls skip school when menstruating, particularly in settings where schools lack adequate WASH facilities [4,9–11]. For example, in Burkina Faso, 11% of schools provide menstrual waste bins in girls’ toilets [12]. In this context, social stigmas and bullying – both in and outside of school – can lead to girls withdrawing from classroom participation, dropping out of school altogether, or experiencing feelings of shame and diminished self-esteem [4,7,13]. Despite these well-documented barriers, there has been limited exploration of how cultural norms within Burkina Faso shape attitudes, and how such attitudes or understandings affect menstruating schoolgirls. This gap in understanding underscores a necessity for a deeper investigation into challenges and cultural dynamics that influence girls’ menstrual hygiene experiences.

Traditional qualitative methods, such as interviews and focus groups, have been invaluable in capturing the lived experiences of women and girls dealing with menstrual health and hygiene challenges [3,14,15]. However, these methods present limitations, especially when applied to adolescents [16]. Adolescents may feel uncomfortable discussing sensitive topics with an interviewer or in group settings, leading to reticence to share genuine feelings [16,17]. Furthermore, reliance on memory during interviews can result in recall bias, which daily reporting methods, such as diaries, may help mitigate [18]. To overcome these limitations, researchers in diverse contexts, including high-income countries (HICs) and low- and middle-income countries (LMICs) are increasingly turning to complementary data sources, including diary methods, as a means to collect data on sensitive topics and to determine whether responses varied across different qualitative techniques [14,19–22].

Diaries, commonly in the form of paper journals, have been utilized in social science and health research [23–27]*.* Whether solicited (where participants or diarists are provided pre-set prompts) or unsolicited (where they record their experience freely), diaries provide participants with a private space to reflect on sensitive experiences, fostering deeper insights over time [28–30]. Diaries have been effective in exploring sensitive or taboo-related topics such as menstruation and HIV in contexts including Malawi, Namibia, and Kenya [14,31–33]. For instance, McLean et al. (2020) used solicited diaries to examine menstrual stigma among schoolgirls in Kenya, demonstrating that diary methods can capture private, day-to-day experiences that may not be readily disclosed in interviews [34]. However, previous evidence also suggests limitations. Cudjoe (2022) in a study in Ghana [35] reported that diaries were perceived as daunting, which contributed to incomplete entries. It is thus unclear whether diaries are a useful data collection method for adolescent girls and sensitive topics.

While diary methods have been used in several settings, to date, no studies have investigated their use to explore menstrual health from the perspective of schoolgirls in Burkina Faso. In our study, we delved into the experiences and challenges of menstrual health and hygiene faced by schoolgirls, and we explored their experiences using a solicited diary method. We further explored whether the depth, detail, and emotional expression of menstruation-related responses differed between diary entries and interviews.

## Methods

### Ethical statement

Our study complied with the 2013 Declaration of Helsinki [36], ensuring the protection of participants’ rights, well-being, and confidentiality. This study was approved by the Institutional Ethics Committee (CIE) of the Centre de Recherche en Santé de Nouna (CRSN), Burkina Faso (Ethics approval: 2018–015-/CIE/ CRSN) and the Medical Faculty of the Ruprecht-Karls University of Heidelberg (Ethics approval: S-654/2018). Prior to data collection, we obtained written informed consent from participants aged 17 and older. For participants under age 17, we secured both informed assent and consent of parents or guardians. Parents or guardians were approached and provided with detailed information about the study objectives and procedures, before their written consent was obtained. Only schoolgirls whose parents or guardians provided consent were invited to provide informed assent and participate in the study. The data were stored and were only accessible to the study team.

### Study design

**Fig 1:**
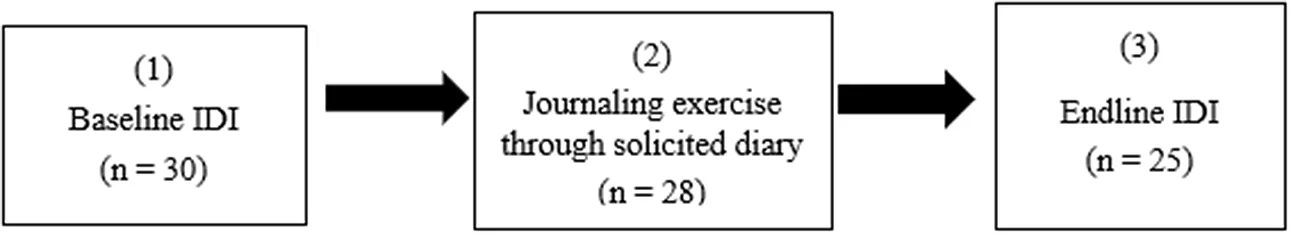
Overview of the study design.

This study was part of a larger menstrual health intervention in Nouna, Burkina Faso with three phases: a baseline phase involving in-depth interviews (IDIs) that were focused on participants’ experiences and perspectives on menstruation and menstrual products distribution; next, an intervention phase (3 months) when youth were given menstrual products to try, and during which they were asked to document their day-to-day menstrual experiences in real time and any changes in their experiences over time using a solicited diary; and finally, endline IDIs, which not only revisited participants’ views on menstruation but also explored their experiences with both menstrual products and the diary-writing process throughout the study (Fig 1). This manuscript focuses on the diaries and IDIs as data sources, and general youth experiences related to menstruation; results specific to the menstrual product intervention will be reported elsewhere.

We decided to try diaries because they allowed participants to record experiences at their own pace and in a familiar environment, potentially reducing social desirability bias often associated with face-to-face interviews. However, given the limited research on diaries as a data collection method among adolescents in similar settings and topics, we were unsure how they would function, particularly given that the topic was sensitive and that the population included girls with a range of literacy levels and comfort with French. We attempted to develop user-friendly diaries by using simple prompts, clear instructions, and simple, age-appropriate French to enhance comprehension, as recommended by previous studies [19,37]. The researchers utilized the Consolidated Criteria for Reporting Qualitative Research (COREQ) to guide the description and reporting of this study [38] (see S1 COREQ Checklist) and the inclusivity statement in attached: S1 Checklist.

### Study setting and sampling

Our study took place in the northwest region of Boucle de Mouhoun in the Kossi Province. This site was chosen due to a longstanding collaboration between the Nouna Health Research Center and the Heidelberg Institute of Global Health, as well as the limited availability of peer-reviewed literature on menstrual health practices in francophone West Africa. With a focus on schoolgirls between 10–19 years, we selected schools that are part of the Nouna Health and Demographic Surveillance System (NHDSS). The NHDSS covers a predominantly rural population with a smaller urban centre (Nouna town), characterized by subsistence farming, limited infrastructure, and diverse ethnic and linguistic groups [39]. However, our sample was limited to urban schools located in Nouna town due to logistical challenges, including long travel distances, seasonal flooding, and poor road conditions. Thus, there is no demographic information available on the urban population from which we sampled our participants. We employed a purposive sampling technique to recruit participants across early to late adolescence (aged 10–19) from seven urban secondary schools in Nouna, including a mix of public (n = 2) and private (n = 5) schools. All schools taught exclusively in French. School administrators identified participants by selecting girls who had reported menstruating and expressed interest in trying the menstrual products distributed in the larger project. Following this, our local research team held an introduction section with the potential participants to explain the objective of the study, consenting process, benefits and risks, and to answer any questions they might have. For participants who were under 17 years of age, our research team and schoolteachers met with their parents/guardians to explain the purpose of the study and obtain consent for their children or wards to be included in the study. Copies of the participant information sheet were provided to schoolgirls and their parents/guardians, who were asked to read the information and make a decision within a 7-day period. After the 7- day window, our research team met with schoolgirls to confirm their interest in participating. Written parental consent and participant assent were then obtained. Schoolgirls who verbally confirmed that they had started menstruating at the time of data collection were recruited into the study. Additionally, their writing literacy was cross-checked through their school subject notebooks to confirm their ability to write basic French. The full list of inclusion and exclusion criteria is attached (see S1 Text).

### Research tools

Our research team developed a semi-structured IDI guides (S2 and S3 Text) and a solicited diary (i.e., with prompts) (S1 File), drawing from a standardized tool developed by UNICEF in 2013 [40]. Both instruments were originally developed in English and then translated into French, the official language used in schools in Burkina Faso. French-speaking research team members conducted translations and reviewed the tools for accuracy.

The IDI guide covered topics such as knowledge of menstruation, societal norms, access to menstrual health and hygiene products, in-school menstrual experience, and in-school WASH facilities. During the first phase of data collection, IDI questions included: “Please tell me words that come to your mind when I say the word *‘menstruation’* ”, “Did you learn about menstruation before having your first period? Who talked to you about it?”. Similarly, questions in the second phase of the IDI included: “Since the last time we met, how would you describe menstrual hygiene management now?”, “Would you use the same words, or do you have more words to describe it?”. During the endline IDIs, participants were also invited to reflect on using a diary over the prior three months, with the question: “How did you feel about the diary?”, “What did you like/dislike about it?”, “What would you change in the diary?”.

The diary employed a paper-based approach, designed with colorful layouts as recommended by previous studies [19,22,41]. The first page of the diary contained instructions for use in French language. Each page of the diary included the same pre-set prompts. Schoolgirls were asked to complete the diaries at home. The diary consisted of 11 questions, seven of which focused on menstrual products (findings related to these products are reported elsewhere) and four of which centered on participants’ overall menstrual experiences, which included: “How did you feel when your period came this month?”.

For the comparative analysis of IDIs versus diaries, we pooled the IDI data from baseline and endline for the four research questions of interest for this study, given that they were not expected to have changed over the course of the longitudinal study. The questions from the IDIs that were analyzed were: “How would you describe your experience?”, “Who do you talk about it with? “If you had new doubts, who did you speak with?” and the questions from the diaries that were analyzed were “Did you speak about this topic with friends at school?”, “What are your ideas to improve the menstrual experience?”. Although the wordings were not identical, these questions sought experiential and emotional responses on how girls felt about menstruation, whom they confided in, and their suggestions for improving menstrual experiences.

Both IDI guides and diaries were piloted with schoolgirls from more urban areas of Nouna, and appropriate refinements, such as adding more space for detailed answers in the diaries, were made. The IDI and diary guides are attached (see Supplementary Material).

### Data collector training

Prior to data collection, six Burkinabé researchers with backgrounds in public health and social science research in Nouna were recruited, each of whom underwent two days of training on qualitative research. This training included comprehensive sessions on research ethics, qualitative data collection methodologies, and best practices for data debriefing. The six researchers served as data collectors for phase 1 and 2 of the IDIs. Furthermore, they were involved in daily debriefing sessions with the research team, wherein emerging themes and areas for further inquiry were identified and discussed.

### Data collection

Interviews were conducted at two time points. In May 2019, we conducted baseline interviews with 30 girls. The interviews with schoolgirls were conducted one-to-one within school premises, in a private room with closed doors provided by the school authority to ensure privacy. Following the completion of these interviews, diaries were distributed, and the girls were guided on how to fill them. By the time of the endline interview three months later, diaries were completed and submitted by 28 of the 30 girls. Participants were informed that participation in the IDI and diary completion was completely voluntary. They were also informed that refusal to participate or discontinuation in the study would not have any influence on any aspect of their lives or school. To ensure full anonymity, participants were encouraged to write in their diaries only when they were alone, thereby promoting privacy and minimizing potential discomfort. Regular follow-ups were also conducted to address any concerns that arose during the diary-keeping process. A final phase of interviews (n = 25) took place three months later in August 2019, where we asked girls to assess their experience using the diary and to provide follow-up information about their menstrual health and hygiene experiences. During the second phase, five participants were unavailable for follow-up interviews because schools in Nouna were closed for summer break, making it difficult to re-establish contact with them. Data saturation was reached for IDIs, as no new information emerged during the later stages of the second round of interviews. Data saturation was not assessed for the diaries because all 30 participants completed them at the same time.

### Data analysis

Interviews were audio recorded and transcribed verbatim into French by experienced data transcribers following qualitative standards [42]. Interview transcripts and diary entries were translated into English and back-translated for quality check and entered into NVivo 12 Pro (QSR International Pty Ltd. Version 12, 2018) for analysis. Thematic analysis was conducted using a combination of inductive and deductive approaches.

First, daily debriefs with the six data collectors enabled an early identification of emerging thematic findings. After all data were collected, we then developed predefined codes based on themes identified during fieldwork debriefs extracted from existing literature [43,44]. Examples of the code included attitude to menstruation, source of information, and period management. Two research team members independently coded the first transcript, followed by an inter-coder alignment session to compare codes. Once the inter-coder alignment was researched, the first author (TA) coded all the transcripts. As new codes or themes emerged from the data, they were applied to the entire dataset. Coded data were reviewed and organized into themes and labelled with a description of their content. To compare findings across the methods, cross-method coding comparisons of four questions were analyzed to explore girls’ emotional and experiential responses. The level of detail and richness in the aggregated codes derived from these questions were compared to examine the extent to which each method captured similar or different responses. Simultaneous review and debriefing sessions ensured consistent data interpretation of diary and IDI data [45].

### Reflexivity

In this study, non-menstruating authors who are male (TA, TB) and non-binary (MDCR) acknowledged an inherent gap in genuinely grasping the lived experiences of menstruating individuals. The collective experience of all authors in previous menstruation-related research provided insights into a more comprehensive questioning of menstrual experiences and challenges. Grounded in constructivist principles, we embraced the notion that reality is subjective, shaped by individual and societal contexts. This epistemological perspective enabled us to consider several viewpoints on menstruation, therefore enhancing our research. The team’s prior research on menstruation informed a nuanced approach to research instruments and facilitated a more comprehensive exploration of the complexities surrounding menstrual experiences.

## Results

IDIs lasted 23–80 minutes, providing data on the girls’ experiences of menarche and menstrual health. The diary exercise lasted three months, with each participant making a minimum of three entries across the entire study period. Each entry averaged between 50 and 120 words, capturing a variety of personal reflections related to menstruation and on diary writing. While our study aimed at recruiting schoolgirls between 10 and 19 years, our final participants’ age range was between 10 and 22 years. Four schoolgirls who were above our initial recruitment age were ultimately recruited because they were actively engaged as students, and the team therefore agreed that their involvement reflected the aim of the research. The mean age of participants in our study is 17.33 years (**± =** 2.99) (See Table 1 below). Our study population was relatively homogeneous, consisting of schoolgirls attending urban public schools with similar socioeconomic backgrounds.

**Table 1:** Demographics characteristics of the study participants (N = 30).

VARIABLES	FREQUENCY (N = 30)	PERCENTAGE (%)
**Age -group (Mean = 17.3 years, Standard Deviation (±) = 2.99, Min = 10, Max = 22)**		
Early Adolescent (10–13 years)	1	3.3
Middle Adolescent (14–17 years)	19	63.3
Late Adolescent (18 – 20 years)	7	23.3
Young Adults (21 years)	3	10.0
**Age of Menarche (Mean = 14.03 years, ± = 1.29, Min = 12, Max = 18)**		
10-13 years	11	36.7
14-16 years	18	60.0
17 years and above	1	3.3

### Primary source of knowledge about menstrual health and hygiene

Most girls reported in IDIs and diaries that their primary source of knowledge about menstrual health and hygiene was their mothers. They mentioned common social meanings associated with menarche, such as the need to “stay away from boys”, the notion of “becoming a woman”, and their mothers’ concerns regarding a risk of pregnancy.

*“(I spoke) with my mother because it is with her that I can speak about things that are about myself. I spoke with her when my first period arrived, and she told me, “Be calm, my child. This is a thing that has meaning for you and other girls, and the meaning is that you are a girl who can have children, and there is no problem. She told me to be faithful to myself with boys” (Girl, 17 years, Diary).*

*“[….] (my mother) said when your period comes, that you put to the idea that you can have children, you must not make a relationship with a boy, you must know that you can give birth, that you have become a woman” (Girl, 16 years, IDI).*

Further insights from the girls revealed that their mothers instilled a strong fear of pregnancy and its potential consequences.

*“She called me to advise me to be wary of boys; otherwise, they can create problems for me. At home, when a girl gets pregnant in her dad's yard, her father banishes her and asks her to leave her class, and my mother insisted a lot on that” (Girl, 18 years, IDI)*

*“[....] (my mother) told me not to be afraid that it is my period that is coming, that I have “become a woman”, that she will tell me, that I should not sleep with a man, that even if I go to sleep with a man that I have to monitor my menstrual cycle” (Girl, 16 years, IDI).*

Upon further probing into what “becoming a woman” meant in their context, girls consistently reaffirmed that becoming a woman implies needing to stay away from boys, and to avoid pregnancy and its consequences (the need to drop out of school, raise children). We further asked girls to describe with whom they speak if they have doubts or follow-up questions related to menstruation and why they choose to speak to that person or group. Extracts from the diary reiterated that girls view their mothers as their most trusted source.

*“… if I had doubts, I would tell my mother. She is the first person, the person whom I trust the most. Because she knows everything in life and equally about all her daughters, and she takes care of us, so I would not tell anybody other than her” (Girl, 17 years, Diary).*

### Socio-cultural context around menstruation

Stigma associated with menstruation leads to constraints such as having to eat alone, avoiding religious spaces, avoiding public gatherings such as festivals, staying away from certain foods, or refraining from conversing with men about periods. These constraints stem from socio-cultural beliefs that menstruating individuals are “dirty” and “disgusting” to people.

*“[..] you must avoid approaching people because you are not clean, some do not accept that you cook. You must be wary of all this. In the public, some people know that you are menstruating, you disgust them, so you must avoid frequenting all these places when you are menstruating.” (Girl, 16 years, IDI)*

Furthermore, participants across Muslim and Christian faiths conveyed that menstruation is perceived as a state of impurity, a view that reflects a stringent interpretation of a religious belief.

*“Because that's dirt.....everything is dirt, God doesn't like dirt.” (Girl, 16 years, IDI)*

*“Among some Muslims, they say that you should not pray if you are on your period, some also say that you should not approach the kitchen” (Girl, 17 years, IDI).*

*“(Among Christians) …there are a lot of things you shouldn't do when you're menstruating. Since if you are on your period, even your prayers are not answered, it is many things that you must not do.” (Girl, 18 years, IDI)*

Some girls described an understanding that praying without the appropriate cleansing during menstruation can be interpreted as an act of defiling God’s name.

*“It is said in Moré, ‘djènab Koné’ among traditional worshippers, that you are disrespecting God or his name. If you want to purify yourself, you must make an incantation before washing yourself, starting with your right side, from the head down, then the left side, and then you can pray; otherwise, the gods will be angry with you” (Girl, 15 years, IDI).*

### In-school menstrual health and hygiene challenges

#### Period bullying and shame.

A major challenge girls described in school is the way they are perceived by others, including peers and teachers. Bullying, especially from boys in school, was described as particularly distressing when menstrual blood leaks onto their school uniforms, amplifying their embarrassment and sense of shame. While some girls acknowledged that certain boys were compassionate, experiences of ridicule from male classmates eclipsed more affirmative exchanges, exacerbating the emotional burden of managing menstruation in a school environment.

*“There are some boys who understand, but others don't; they don't care. That is all their concern. Even in the city, if they are in the company of their friends and they see you passing by, they talk about it until you hear them. They mock you across the city, saying that you can't take care of yourself, at school, your period flows in everyone's eyes. So, anyone who has the information will be eyeing you in the cit. It's shameful. That's how boys are.” (Girl, 15 years, IDI).*

Bullying extended beyond the school walls, with girls sometimes facing insults from male classmates even in public settings.

*“They laugh, they insult you, others may even cross you one day in town and talk to you again, some even point out to you that it is this day your clothes were stained” (Girl, 15 years, IDI).*

While male classmates were described as the most relentless perpetrators of bullying, female classmates and teachers were also described as shaming girls who could not adequately hide their menstruation. In interviews and diaries, girls described how female classmates and teachers lacked empathy and viewed stained clothing as a personal failing or an outward indication of a girl’s inability to “take care of oneself”.

#### Academic performance and concentration.

A fear of soiling uniforms weighed heavily on girls, leading to anxiety and diminished focus in class. This distraction occasionally interfered with girls’ ability to concentrate or to respond to questions posed by the teacher in class (as responding to teachers typically requires a student to stand up, and thus potentially reveal her soiled clothing). Girls described feeling humiliated and fearing punishment.

*“You can't concentrate anymore because you're afraid. You don't know if you're stained, and it's complicated... if you are in class, you are not happy, because if the teacher questions you suddenly, and if you refuse to get up, he will not understand, and if you are not careful, he can punish you because of this behavior” (Girl, 17 years, IDI).*

#### Inadequate WASH facilities.

Girls in our study highlighted inadequacies of WASH facilities, stressing a need for clean and well-maintained toilets, private and well-equipped changing rooms, and easy access to clean water and soap. In one school, where two or three toilets are shared between boys and girls, these challenges are especially significant for menstruating girls.

*“You also need a discreet place to throw away your used pads. Inside the toilets, you find everything wet with urine, even the walls, so you don’t even want to go in. It’s not easy.” (Girl, 17 years, IDI)*

Another participant highlighted privacy concerns associated with inadequate facilities, leaving girls vulnerable to embarrassment and discomfort, especially while changing sanitary pads.

*“It’s not that simple because our toilets are used by both girls and boys. If you go in to change and you’re not careful, a boy could walk in on you—it’s complicated.” (Girl, 17 years, IDI)*

Study participants further suggested ways to improve WASH facilities, specifically focusing on improving menstrual health and hygiene in schools. Some of the girls suggested an urgent need for gender-sensitive toilets in schools.

*“If we could have separate facilities for girls, completely away from the boys’ area, it would prevent any contact. Some boys can be quite rude and try to peek inside. It would also be helpful if we could have showers in the toilets.” (Girl, 16 years, IDI)*

Girls’ suggestions reflect their desire for improved WASH facilities and demonstrate their advocacy for younger students, particularly their sisters, who may face similar challenges in the future.

*“[…] building a little house in our school where we can find pads, water, a place where we girls can change, clean ourselves, and wear another pad when we are on our period. God would bless you for such a great gesture, and we would recognize (thank) you for that. Even if you are not going to do it for us, do it for our little sisters, so that they do not suffer like us. Please?” (Girl, 16 years, Diary)*

### Diary experience: participants’ perspective

Beyond exploring menstrual health and hygiene, our qualitative data also captured girls’ shared experiences using diaries as a research tool (see Table 2). Girls reported that diaries enabled them to articulate their thoughts, express themselves freely, share their menstruation stories, and engage with a taboo topic in a private manner. Furthermore, girls likened diary writing to an experiment that allowed them to explore their ideas. Diary prompts about menarche and menstrual materials particularly resonated with girls, indicating that these topics held palpable interest. However, girls faced challenges with written French and found the repeated monthly questions uninteresting because their reflections did not change over the 3-month period.

**Table 2:** Participants’ experience with the diary.

Category	Sub-category	Description	Example
**Facilitators to diary writing**	Space for ideas and self-expression	Participants valued the opportunity to express their thoughts, ideas, and experiences freely through their diary entries, underscoring the role of diary writing as a tool for personal expression and reflection.	*“…it allows me to express myself and say everything I think about the project” (Girl, 15 years, Diary).* *“I liked it because I expressed myself about what I experienced. Because you express yourself, you say your ideas, your personal thoughts, so you express everything. It allowed us to say what we ourselves think about menstruation, to free ourselves, or to tell stories about our periods, and in the second place it allowed us to know about menstruation” (Girl, 22 years, Diary).*
	Increasing familiarity and comfort	Participants’ acceptance of the journaling task evolved over time. Initial discomfort and reluctance, while not uncommon, gradually gave way to eventual acceptance.	*“Well, in the first month anyway, I was ashamed to write something, but over time I got used to it” (Girl, 15 years, Diary).*
	Opportunity to engage with a taboo topic	Participants highlighted their appreciation for the questions on menarche and types of menstrual materials, emphasizing how the diary provided a valuable space for self-reflection on these often-taboo subjects.	*“What I liked were the parts about, at what age did you have your first period and how it went, if you suffered from it or not. That's what I liked about this diary” (Girl, 15 years, Diary).*
	Privacy	The diary acted as a safe sanctuary, allowing participants to express themselves freely without the fear of judgment or scrutiny from others.	*“I felt good, you don't need to tell your comments to your friends, you can write it in the diary and feel comfortable, and only you know about it” (Girl, 15 years, Diary).*
**Barriers to diary writing**	Limitations of language proficiency and writing	A participant’s ability to respond to the diary questions was heavily influenced by their proficiency in the French language and their writing skills. While many of the girls spoke French, some found written French to be a limiting factor. For some participants, the questions were straightforward, but others struggled to understand with the complexity of certain prompts, making it challenging to fully express their thoughts.	*“…the part of ideas and thoughts you have to look for words and formulate with your own French, look for your words, that's not easy” (Girl, 18 years, Diary).* *“French was a bit difficult to understand” (Girl, 15 years, Diary).* *“I would like the questions to be asked in simple French, so that the words are not too complicated to understand” (Girl, 15 years, Diary).* *“The questions weren't difficult, but I was the one who didn't understand. As you also explained there, it's not complicated, if nothing else” (Girl, 18 years, Diary).*
	Added workload	Some participants stated that the exercise resembled being assigned a task, as responding to the diary questions required dedicated time for eliciting deeper observations.	*“It's like I have been given work, because to answer questions, you have to sit down and think, all by yourself, to give answers and keep thinking. There are questions that I did not understand” (Girl, 18 years, Diary).* *“Filling out this diary was very complicated for me, because it had difficult questions to answer” (Girl, 15 years, Diary).*
	Perceived repetition due to probing questions	Our data collection team attempted to draw more information through probing questions. However, participants perceived this approach as repetition.	*“It's the questions that are repeated, you don't know how to answer, you answer here, you turn the page, it's pretty much the same question” (Girl, 22 years, Diary).* *“There are questions that are repeated, that you have already answered, and you do not know what to write. You won't be able to write much anymore because you've said everything before” (Girl, 16 years, Diary).*

### Comparison of IDI and diary methods

Using a cross-method coding comparison approach, there was no consistent trend in the similarities or differences in responses to the four comparable questions across the two qualitative methods. For most participants, the level of detail and emotional expression in the diary entries was comparable to that of the IDIs, suggesting that the emotional depth and content in both methods were primarily shaped by individual participant responses rather than by the method itself (see Table 3 below).

**Table 3:** Methodological rigor and code detail across IDIs and diaries.

Code	IDIs	Diaries
Fear and confusion at menarche	+++	+++
Menstrual pain and physical discomfort	+++	++
Source of information	+++	+++
Shame	++	+
Peer discussion and knowledge exchange	+	++
Confidence/readiness	+	+++
Coping strategies	+++	++
Suggestions for improving menstrual experiences	+++	+++

Key: +++ = rich and detailed data; ++ = moderate detail; + = *limited or brief detail.*

In Table 4, we compared the four menstruation-related questions in the diary entries with those in the IDIs, highlighting how the two methods compared across different participants and topics.

**Table 4:** Comparison of IDI and diary entries among participants.

Questions/prompts	Response in IDI	Response in Diary	Observation
How did you feel when your period came? How would you describe your experience?	*“I saw this in class, I myself did not know, it was when I arrived home, I saw blood and my stomach started to hurt, I said to myself, but what is this? That's how I told my mother, and she has now explained to me what it was.” (Girl, 17 years, IDI)* *“On the first day of my period, when I got up in the morning, I couldn't walk. That day, I woke up very early, and I had stomach aches until nine o'clock, and my period… it was only the next day that the stomach aches started. That's how it happened.” (Girl, 22 years, IDI)*	*“The day I saw my first period, I was afraid (I had fear), I had never seen that. I asked my mother what was that, and my mother responded that it was my period, and I still asked how to do to make it end, and my mother told me there was nothing to do to make it end. Then my mother gave me the money to go buy (pad brand). When I returned home, she showed me how to protect myself. After that, even if my period comes, I do not need to ask how to protect myself” (Girl, 17 years, Diary).* *“When my period came, I felt comfortable and ready. During this second month, I would describe my experience: I was a very proud girl, and my period was not bothering me anymore. I was free like I wanted.” (Girl, 22 years, Diary)*	Though similar in content, the diary entry reflects a more positive emotional state and a sense of readiness, while the IDI response describes discomfort and the progression of symptoms.
If you had new doubts, who did you speak with? Why?	*“It was when my period came that I got the information. In any case, it was my mother who gave me the information… I didn't want anyone to know, but in the end, I went to tell her, I couldn't get close to my father because I was ashamed of this very thing… She is a woman and knows how it goes, she has the experience and can give you advice on that, on her evolution, that's why I approach her for advice, and also, I'm ashamed to talk to my father about it.” (Girl, 17 years, IDI)*	*“(I spoke) with my mother because it is with her that I can speak about things that are about myself. I spoke with her when my first period arrived and she told me, “be calm, my child. This is a thing that has meaning for you and other girls, and the meaning is that you are a girl that can have children and there is no problem (illness scratched)”. She told me to be faithful to myself with boys” (Girl, 17 years, Diary).*	Both responses are rich in content. We noted no particular difference in response.
Did you speak about this topic with friends at school?	*“No, I don’t know, talking about it is embarrassing…It was the same as the others.” Girl, 15 years, IDI)*	*“Yes, I spoke about this topic with friends at school, they told me that when their period comes, they have abdominal pain. Another one told me something else. Yes, I heard my classmates speak about this topic or about the project. They said that if you have seen your period (if your menarche has arrived), you have to pay attention because you can quickly fall pregnant.” (Girl, 15 years, Diary)*	The IDI response indicates embarrassment, whereas the diary entry reflects a more positive tone, showing more engagement and information exchange among peers. In future cases, this discrepancy would highlight an avenue for further probing.
What are your ideas to improve the menstrual experience?	*“We can talk between girls outside of school hours, to share experiences, you can ask a girl how she will manage if her period occurs while she is in the middle of class, she can tell you that if you have a classmate you can ask her if she has pads or pieces of loincloths in her, and she can give you to protect yourself. But before you get up, if the skirt is stained, as we have black skirts, you can turn the skirt to the side and go out to wear your diapers.” (Girl, 18 years, IDI)*	*“I would like to address girls here that we should not be afraid of speaking about menstruation to our mothers because if we hide the day, we will have problems. I will be too late at that point/moment. Girls should trust their mothers. In case of these types of things, they should not be afraid of speaking to their parents.” (Girl, 18 years, Diary)*	Both responses suggest improvements, but from different perspectives: practical strategies versus communication and trust.

## Discussion

### Menstrual health and hygiene experience and challenges

Our study highlights (a) how socio-cultural factors and classroom settings shape girls’ menstrual experiences, and (b) how girls engage with diaries and perceive their utility as a component of qualitative research. On the former point, girls in our study described receiving information on menstruation primarily from their mothers and typically framed around pregnancy risk and a need to avoid boys. Major challenges faced as a menstruating person include cultural and religious restrictions, in-school bullying, and limited WASH facilities. On the latter point, the diary was described as a safe space for girls to narrate their experiences and engage in self-expression, although limited writing skills and perceived repetitive questioning were reported.

Similar to findings from a comprehensive review and previous studies conducted in Jordan and The Gambia, most girls reported that their primary source of information about menarche was their mothers [46–48]. However, unlike studies in Honduras, Tanzania, Ghana, and India, where mothers were found to provide robust information [49–52], our findings indicate a relatively limited presentation of the topic. For instance, information such as “(Menstruation) means you are a woman, you have to stay away from boys, you can get pregnant”, largely reflects a fear or risk-infused message largely tied to sexuality [53,54]. Findings from a comparable qualitative study in Ethiopia reported that girls are deemed mature for sexual activities once they reach menarche, and such understandings reinforce early sexual debut and out-of-wedlock pregnancies [53]. Considering the rising rates of teenage pregnancy in Burkina Faso in 2022, which surpassed average rates observed in similar low-income countries [55], it is increasingly pertinent to emphasize a need for comprehensive information and support for both girls and their mothers. Challenging negative perceptions of menstruation and fostering open conversations about sexual health remain equally crucial [56–58].

In Burkina Faso, socio-cultural contexts relating to restrictions and stigma remain a major challenge for adolescent girls, hindering existing efforts to improve menstrual health and hygiene practices [7,13]. Our findings resonate with studies conducted in Scotland and The Gambia, which demonstrate that socio-cultural contexts play significant roles in shaping overall menstrual experiences [48,59]. Similar observations were found in Nigeria and Kenya, where menstruating girls and women are prohibited from performing basic household chores, such as cooking [14], and are discouraged from entering public places [60]. Such practices contribute to a stigmatization of menstruating individuals [46,61].

Religious restrictions further exacerbate challenges surrounding menstruation. For instance, some Muslim participants reported that they are prohibited from praying while menstruating, as menstrual blood is considered dirty [54]. However, this religious restriction is not exclusive to the Muslim community [7,53]. Other religious groups, including indigenous worshippers, shared similar beliefs, with some considering menstruating girls as potentially angering the gods [62,63]. Similarly, a qualitative study reported that women in Fiji, the Solomon Islands, and Papua New Guinea are excluded from participating in religious activities within Christian and Hindu faiths [63]. There is a prevailing belief that girls must undergo ritual cleansing before they can engage in prayers [62]. Adopting a faith-based approach may prove insightful to mitigate restrictions. Studies have shown that religious leaders hold a prominent position and voice in their communities [64–67]. Hence, engaging them may be crucial in facilitating a shift in the negative cultural narratives surrounding menstruation [64,67].

Bullying and inadequate WASH facilities proved to be major challenges faced by Burkinabe schoolgirls. Similar findings have been reported from Tanzania and the USA, indicating that girls experience awkward gestures, name-calling, and bullying related to menstruation, suggesting that period bullying is prevalent in schools [68,69]. When subjected to period bullying, girls may experience heightened feelings of shame, guilt, and embarrassment [10]. Our findings also echoed the results from studies in Kenya, Uganda, and Tanzania, which revealed that schoolgirls reported feelings of embarrassment stemming from comments, jokes, and gestures, especially from schoolboys, regarding menstrual leaks or stains [11,70–73]. These experiences further fueled their feelings of guilt and fear of becoming the target of ridicule among their peers in school [71–73]. Continuous bullying, both within and beyond school settings, can significantly impact girls’ participation and engagement in various aspects of school life [11,72].

In contrast to a study in Kenya, which found adequate facilities for menstrual health and hygiene practice in schools [74], our participants reported inadequacies in WASH facilities, especially concerning the availability of soap, locks on bathroom doors, and water [12,75]. These findings align with another study in Uganda, which emphasized a persistent lack of infrastructural and hygiene facilities, including an absence of private toilets for changing and washing, despite several interventions in schools [76]. The absence of a safe and hygienic environment to address menstrual needs exacerbates girls’ discomfort and contributes to absenteeism- a detrimental consequence of inadequate WASH facilities [11,77–79]. Drawing from the results and other LMICs, our study underscores a critical need to implement comprehensive programs in schools that address WASH infrastructure, menstrual health education, and anti-bullying and bullying initiatives. Such efforts should also engage men and boys, recognizing their role in shaping gender norms and their influence on menstrual experiences [80,81]. Furthermore, a collaborative dialogue that engages girls, parents, religious leaders, teachers, and the wider community is essential to creating a supportive environment that empowers girls and fosters their confidence in and out of school [54,82,83].

### Diary insights: participants’ experience, and comparison with IDIs

There was no common consensus as to how the girls perceived the diary. For some, the diary writing exercise allowed space for self-expression and the articulation of personal ideas and thoughts, which reflects reports from a book chapter and previous qualitative studies in Turkey and Indonesia [24,84,85]. In this sense, diaries can be a tool for personal expression. Through diaries, participants in our study felt a sense of ownership over their narratives, allowing them to share their experiences at their own pace [86,87]. Similar to a qualitative study in the United Kingdom on young people’s alcohol consumption experiences, diaries facilitated engagement with a taboo topic [88], creating a private space to be candid and alleviating feelings of embarrassment or fear of judgment [23,88,89].

In our study, some participants struggled to articulate their thoughts in written French - a challenge that was not identified during the pilot phase of this study. Limited language proficiency and writing skills may lead to feelings of frustration and inhibit participant engagement in diary use [90]. Previous research has documented how such challenges may foster feelings of frustration and uncertainty about providing new insights [41,91,37], which underscores the importance of reviewing the diaries at distribution to clarify perceived repetitive or difficult questions [20,22]. Researchers may also explore alternative methods suited to participants’ needs, such as audio diaries, among young people with limited language and writing skills [26,92,93].

When comparing diary entries to IDIs, we observed no consistent differences in responses across the two methods, which suggests that both methods can yield comparable levels of detail and depth in participants’ narratives [94–96]. However, the similarity in content and details we observed in our study may be context-specific and may not necessarily apply to other settings. For instance, qualitative studies in the UK and Kenya reported that diaries provided more detailed and in-depth responses compared to verbal inquiries alone [14,97]. Hence, future research should consider which method, or combination of methods, works best in their specific context.

During the development of the study and diary, we anticipated that a three-month period would allow us to capture differences in daily records and the everyday experiences of menstruation-related challenges among schoolgirls. Despite this period of daily recording, their entries did not show notable differences, which may indicate that, in this study, longitudinal recounting may not have provided additional insights. Diaries were not as effective as we had anticipated for exploring sensitive topics such as menstruation among adolescent girls, given that they produced findings similar to those generated through IDIs. Using diaries in low-resource school settings demands extensive effort from the research team, including the provision of clear guidance and regular follow-up or reminders. Future qualitative research in similar contexts should consider potential limitations, including literacy barriers, limited writing skills, writing fatigue, self-censorship, and incomplete entries.

### Strengths and limitations

By employing solicited diaries and IDIs, our study provided valuable insights into the lived experiences and challenges related to menstrual health and hygiene among schoolgirls in Burkina Faso. Additionally, our study is the first research that explored menstrual health and hygiene utilizing diaries for research in Burkina Faso. However, our sample was limited to urban schools, as logistical difficulties, such as long travel times, flooding, and muddy roads during the rainy season, prevented access to rural areas. Consequently, our findings may not fully represent the experiences of schoolgirls in rural areas. Although younger participants (under 15 years) were encouraged to share their experiences, quotations from our study were primarily from older participants because their accounts were perceived as clearer and richer. This may have introduced bias and underrepresented younger girls’ perspectives, particularly as differences in quotations between the two age groups were not analyzed. In selecting participants for diary writing, we cross-checked writing and literacy requirements, which may have excluded other schoolgirls with different experiences. Additionally, most menstruation-related questions were asked specifically in the IDIs and not included in the diary prompts. Consequently, only four comparable questions could be examined across both methods, meaning that most themes were informed by one method, which limited adequate comparability of the results. Participants’ experiences with using different types of menstrual products, comfort, and usability from the larger study will be captured in a separate publication. We encourage further research to explore the menstrual experience of schoolgirls in rural areas of Burkina Faso and beyond.

## Conclusion

Our study highlights that schoolgirls received limited information on menstruation framed by fear of pregnancy and avoiding boys. These misconceptions, alongside in-school bullying, sociocultural and religious restrictions, and inadequate WASH facilities, negatively impact their menstrual health experiences. To address these challenges, policy efforts should focus on improving knowledge and directly confronting cultural and religious barriers that perpetuate menstrual stigma. These efforts require meaningful engagement not only with girls themselves but also with key stakeholders, including school authorities, parents, government representatives, community elders, and religious and spiritual leaders [98,99]. Co-developing girl-led, gender-sensitive WASH facilities that ensure privacy, dignity, and safety, alongside integrating menstrual health education into school curricula, are essential steps toward transforming the overall menstrual experience. While diaries may add more content and depth than IDIs in other settings, the methods captured similar level of detail and richness across the four comparable questions in our study, suggesting that a multi-method approach may not be necessary for capturing rich qualitative insights within our study context on this topic. As interest in alternative data collection methods such as diaries continues to grow, further studies may explore the use of audio diaries in settings where writing skills and language proficiency pose challenges.

## Supporting information

S1 COREQ Checklist COREQ (COnsolidated criteria for REporting Qualitative research) Checklist. (PDF)

S1 Checklist Inclusivity in global research. (DOCX)

S1 Text Inclusion criteria. (DOCX)

S2 Text Interview guide phase 1. (DOCX)

S3 Text Interview guide phase 2. (DOCX)

S1 File Diary. (PPTX)

## Abbreviations

CRSN: Centre de Recherche en Santé de Nouna
IDI: in-depth interview
CIE: institutional ethics committee
NHDSS: Nouna health and demographic surveillance site
WASH: water, sanitation, and hygiene

## References

[1] Kuhlmann AS, Henry K, Wall LL. Menstrual Hygiene Management in Resource-Poor Countries. Obstet Gynecol Surv. 2017;72(6):356–76. doi: 10.1097/OGX.0000000000000443 28661550 PMC5482567

[2] Hennegan J, Montgomery P. Do Menstrual Hygiene Management Interventions Improve Education and Psychosocial Outcomes for Women and Girls in Low and Middle Income Countries? A Systematic Review. PLoS One. 2016;11(2):e0146985. doi: 10.1371/journal.pone.0146985 26862750 PMC4749306

[3] Baku EA, Adrakpanya V, Konlan KD, Adatara P. Menstrual hygiene management among girls at a peri-urban senior high school in the Volta Region, Ghana. Afr J Midwifery Women’s Health. 2020;14(1):1–12. doi: 10.12968/ajmw.2018.0020

[4] Keihas L. Menstrual hygiene in schools in two countries of francophone West Africa: Burkina Faso and Niger. Case studies in 2013 | Health and Education Resource Centre [Internet]. 2013 [cited 2024 Jul 10]. Available from: https://healtheducationresources.unesco.org/library/documents/menstrual-hygiene-schools-two-countries-francophone-west-africa-burkina-faso-and

[5] Phillips-Howard PA, Caruso B, Torondel B, Zulaika G, Sahin M, Sommer M. Menstrual hygiene management among adolescent schoolgirls in low- and middle-income countries: research priorities. Glob Health Action. 2016;9:33032. doi: 10.3402/gha.v9.33032 27938648 PMC5148805

[6] Performance Monitoring and Accountability. PMA2020-Menstrual Hygiene Management BURKINA FASO. 2017.

[7] Krenz A, Strulik H. The impact of menstruation hygiene management on work absenteeism of women in Burkina Faso. Econ Hum Biol. 2021;43:101067. doi: 10.1016/j.ehb.2021.101067 34655853

[8] Das P, Baker KK, Dutta A, Swain T, Sahoo S, Das BS, et al. Menstrual Hygiene Practices, WASH Access and the Risk of Urogenital Infection in Women from Odisha, India. PloS One. 2015;10(6):e0130777. doi: 10.1371/journal.pone.0130777 26125184 PMC4488331

[9] Fehr AET. Stress, menstruation and school attendance: effects of water security on adolescent girls in South Gondar, Ethiopia. 2010.

[10] McMahon SA, Winch PJ, Caruso BA, Obure AF, Ogutu EA, Ochari IA, et al. “The girl with her period is the one to hang her head” Reflections on menstrual management among schoolgirls in rural Kenya. BMC Int Health Hum Rights. 2011;11:7. doi: 10.1186/1472-698X-11-7 21679414 PMC3129305

[11] Sedekia Y, Kapiga S, Mcharo O, Luwayi J, Sasi SK, Tanton C, et al. “I know your problems; take your bag and go home”: a qualitative study using the social-ecological model to understand drivers of suboptimal school and social participation among secondary schoolgirls in Northwest Tanzania. BMC Public Health. 2025;25(1):1969. doi: 10.1186/s12889-025-23101-8 40437413 PMC12117854

[12] UNICEF, WHO. Progress on drinking water, sanitation and hygiene in schools 2015–2023: special focus on menstrual health. 2024.

[13] Kabore I, Nikiema LZP, Debus JP, McIntosh C. Menstrual hygiene management to improve the attendance of primary school-aged girls in Central North, Burkina Faso. 2019.

[14] MacLean K, Hearle C, Ruwanpura KN. Stigma of staining? Negotiating menstrual taboos amongst young women in Kenya. Women’s Stud Int Forum. 2020;78:102290. doi: 10.1016/j.wsif.2019.102290

[15] Morrison J, Basnet M, Bhatt A, Khimbanjar S, Chaulagain S, Sah N, et al. Girls’ Menstrual Management in Five Districts of Nepal: Implications for Policy and Practice. Stud Soc Justice. 2018;12(2):251–72. doi: 10.26522/ssj.v12i2.1623

[16] Dashiff C. Data collection with adolescents. J Adv Nurs. 2001;33(3):343–9. doi: 10.1046/j.1365-2648.2001.01670.x 11251721

[17] Koning IM, Harakeh Z, Engels RCME, Vollebergh WAM. A comparison of self-reported alcohol use measures by early adolescents: Questionnaires versus diary. J Subst Use. 2010;15(3):166–73. doi: 10.3109/14659890903013091

[18] Zurbriggen CLA, Jendryczko D, Nussbeck FW. Rosy or blue? Change in recall bias of students’ affective experiences during early adolescence. Emotion. 2021;21(8):1637–49. doi: 10.1037/emo0001031 34928636

[19] Akinreni T, Reñosa MDC, Putri AZ, Scott K, McMahon SA. A methodological review of solicited diaries as a qualitative tool in health research in low- and middle-income countries. SSM - Qual Res Health. 2024;6:100492. doi: 10.1016/j.ssmqr.2024.100492

[20] Munyewende PO, Rispel LC. Using diaries to explore the work experiences of primary health care nursing managers in two South African provinces. Glob Health Action. 2014;7:25323. doi: 10.3402/gha.v7.25323 25537937 PMC4275646

[21] O’Reilly K, Ramanaik S, Story WT, Gnanaselvam NA, Baker KK, Cunningham LT, et al. Audio Diaries: A Novel Method for Water, Sanitation, and Hygiene-Related Maternal Stress Research. Int J Qual Methods. 2022;21:160940692110732. doi: 10.1177/16094069211073222

[22] Tripathy S, Acharya SP, Sahoo AK, Mitra JK, Goel K, Ahmad SR, et al. Intensive care unit (ICU) diaries and the experiences of patients’ families: a grounded theory approach in a lower middle-income country (LMIC). J Patient Rep Outcomes. 2020;4(1):63. doi: 10.1186/s41687-020-00229-2 32705412 PMC7378135

[23] Alaszewski A. Diaries as a source of suffering narratives: A critical commentary. Health Risk Soc. 2006;8(1):43–58. doi: 10.1080/13698570500532553

[24] Dincel BK, Savur H. Diary Keeping in Writing Education. J Educ Train Stud. 2018;7(1):48. doi: 10.11114/jets.v7i1.3758

[25] Ewijk CD van, Fabriz S, Büttner G. Fostering self-regulated learning among students by means of an electronic learning diary: A training experiment. J Cogn Educ Psychol. 2015;14(1):77–97. doi: 10.1891/1945-8959.14.1.77

[26] Worth N. Making Use of Audio Diaries in Research with Young People: Examining Narrative, Participation and Audience. Sociol Res Online. 2009;14(4):77–87. doi: 10.5153/sro.1967

[27] Sargeant S, Gross H. Young people learning to live with inflammatory bowel disease: working with an “unclosed” diary. Qual Health Res. 2011;21(10):1360–70. doi: 10.1177/1049732311407211 21525239

[28] Adler A, Gujar A, Harrison BL, O’Hara K, Sellen A. A diary study of work-related reading: design implications for digital reading devices. In: Proceedings of the SIGCHI conference on Human factors in computing systems - CHI ’98 [Internet]. Los Angeles (CA): ACM Press; 1998 [cited 2023 Mar 23]. p. 241–8. Available from: http://portal.acm.org/citation.cfm?doid=274644.274679

[29] Jones RK. The unsolicited diary as a qualitative research tool for advanced research capacity in the field of health and illness. Qual Health Res. 2000;10(4):555–67. doi: 10.1177/104973200129118543 11010078

[30] Meth P. Entries and omissions: using solicited diaries in geographical research. Area. 2003;35(2):195–205. doi: 10.1111/1475-4762.00263

[31] Bailey SL, Gao W, Clark DB. Diary study of substance use and unsafe sex among adolescents with substance use disorders. J Adolesc Health. 2006;38(3):297.e13–e20. doi: 10.1016/j.jadohealth.2004.12.001 16488830

[32] Mkandawire AK, Jumbe V, Nyondo-Mipando AL. To disclose or not: experiences of HIV infected pregnant women in disclosing their HIV status to their male sexual partners in Blantyre, Malawi. BMC Public Health. 2022;22(1):1552. doi: 10.1186/s12889-022-13974-4 35971103 PMC9377067

[33] Thomas F. Stigma, fatigue and social breakdown: exploring the impacts of HIV/AIDS on patient and carer well-being in the Caprivi Region, Namibia. Soc Sci Med. 2006;63(12):3174–87. doi: 10.1016/j.socscimed.2006.08.016 16987574

[34] MacLean K, Hearle C, Ruwanpura KN. Stigma of staining? Negotiating menstrual taboos amongst young women in Kenya. Women’s Stud Int Forum. 2020;78:102290. doi: 10.1016/j.wsif.2019.102290

[35] Cudjoe E. Using diaries with interpretative phenomenological analysis: Guidelines from a study of children whose parents have mental illness. Int J Qual Methods. 2022;21:16094069221084435. doi: 10.1177/16094069221084435

[36] World Medical Association. World Medical Association Declaration of Helsinki: Ethical Principles for Medical Research Involving Human Subjects | Research, Methods, Statistics | JAMA | JAMA Network [Internet]. 2013 [cited 2024 Dec 9]. Available from: https://jamanetwork.com/journals/jama/fullarticle/1760318 10.1001/jama.2013.281053 24141714

[37] Janssens KAM, Bos EH, Rosmalen JGM, Wichers MC, Riese H. A qualitative approach to guide choices for designing a diary study. BMC Med Res Methodol. 2018;18(1):140. doi: 10.1186/s12874-018-0579-6 30445926 PMC6240196

[38] Tong A, Sainsbury P, Craig J. Consolidated criteria for reporting qualitative research (COREQ): a 32-item checklist for interviews and focus groups. Int J Qual Health Care. 2007;19(6):349–57. doi: 10.1093/intqhc/mzm042 17872937

[39] Sié A, Louis VR, Gbangou A, Müller O, Niamba L, Stieglbauer G, et al. The Health and Demographic Surveillance System (HDSS) in Nouna, Burkina Faso, 1993–2007. Glob Health Action. 2010;3:10.3402/gha.v3i0.5284. doi: 10.3402/gha.v3i0.5284 20847837 PMC2940452

[40] Caruso B. WASH in Schools Empowers Girls’ Education: Tools for Assessing Menstrual Hygiene Management in Schools [Internet]. United Nations Children’s Fund (UNICEF); 2014 [cited 2024 Aug 7]. Available from: https://www.researchgate.net/publication/270880911_WASH_in_Schools_Empowers_Girls’_Education_Tools_for_Assessing_Menstrual_Hygiene_Management_in_Schools

[41] Cudjoe E. Using diaries with interpretative phenomenological analysis: guidelines from a study of children whose parents have mental illness. Int J Qual Methods. 2022;21:160940692210844. doi: 10.1177/16094069221084435

[42] McLellan E, MacQueen KM, Neidig JL. Beyond the Qualitative Interview: Data Preparation and Transcription. Field Methods. 2003;15(1):63–84. doi: 10.1177/1525822x02239573

[43] Hennegan J, Brooks DJ, Schwab KJ, Melendez-Torres GJ. Measurement in the study of menstrual health and hygiene: A systematic review and audit. PLoS One. 2020;15(6):e0232935. doi: 10.1371/journal.pone.0232935 32497117 PMC7272008

[44] Hennegan J, Nansubuga A, Smith C, Redshaw M, Akullo A, Schwab KJ. Measuring menstrual hygiene experience: development and validation of the Menstrual Practice Needs Scale (MPNS-36) in Soroti, Uganda. BMJ Open. 2020;10(2):e034461. doi: 10.1136/bmjopen-2019-034461 32071187 PMC7044919

[45] McMahon SA, Winch PJ. Systematic debriefing after qualitative encounters: an essential analysis step in applied qualitative research. BMJ Glob Health. 2018;3(5):e000837. doi: 10.1136/bmjgh-2018-000837 30233833 PMC6135453

[46] Chandra-Mouli V, Patel SV. Mapping the knowledge and understanding of menarche, menstrual hygiene and menstrual health among adolescent girls in low- and middle-income countries. In: Bobel C, Winkler IT, Fahs B, Hasson KA, Kissling EA, Roberts TA, editors. The Palgrave Handbook of Critical Menstruation Studies. Singapore: Palgrave Macmillan; 2020. 33347167

[47] Jarrah SS, Kamel AA. Attitudes and practices of school-aged girls towards menstruation. Int J Nurs Pract. 2012;18(3):308–15. doi: 10.1111/j.1440-172X.2012.02032.x 22621303

[48] Shah V, Nabwera HM, Sosseh F, Jallow Y, Comma E, Keita O, et al. A rite of passage: a mixed methodology study about knowledge, perceptions and practices of menstrual hygiene management in rural Gambia. BMC Public Health. 2019;19(1):277. doi: 10.1186/s12889-019-6599-2 30845945 PMC6407285

[49] Accerenzi M, Brañas-Garza P, Jorrat D. Parents’ knowledge and predictions about the age of menarche: experimental evidence from Honduras. Arch Public Health. 2023;81(1):20. doi: 10.1186/s13690-023-01030-5 36765423 PMC9912583

[50] Njee RM, Imeda CP, Ali SM, Mushi AK, Mbata DD, Kapala AW, et al. Menstrual health and hygiene knowledge among post menarche adolescent school girls in urban and rural Tanzania. PLoS One. 2024;19(3):e0284072. doi: 10.1371/journal.pone.0284072 38466719 PMC10927101

[51] Sudhakar BP, R SS. A cross sectional study of knowledge and practices about reproductive health among female adolescents in an urban slum of Mumbai. 2011;5(4):119–26.

[52] Mohammed S, Larsen-Reindorf RE. Menstrual knowledge, sociocultural restrictions, and barriers to menstrual hygiene management in Ghana: Evidence from a multi-method survey among adolescent schoolgirls and schoolboys. PLoS One. 2020;15(10):e0241106. doi: 10.1371/journal.pone.0241106 33091080 PMC7580927

[53] Betsu BD, Medhanyie AA, Gebrehiwet TG, Wall LL. “Menstruation is a Fearful Thing”: A Qualitative Exploration of Menstrual Experiences and Sources of Information About Menstruation Among Adolescent Schoolgirls. Int J Womens Health. 2023;15:881–92. doi: 10.2147/IJWH.S407455 37283993 PMC10239630

[54] Chew KS, Wong SSL, Hassan AK, Po KE, Zulkhairi N, Yusman NAL. Socio-Cultural and Religious Influences During Menstruation Among University Students [preprint] [Internet]. In Review; 2020&nbsp;[cited 2023 Jun 20]. Available from: https://www.researchsquare.com/article/rs-32211/v1

[55] World Bank Group. World Bank Gender Data Portal [Internet]. Burkina Faso; 2022 [cited 2024 Dec 9]. Available from: https://liveprod.worldbank.org/en/economies/burkina-faso

[56] Crawford M, Menger LM, Kaufman MR. “This is a natural process”: managing menstrual stigma in Nepal. Cult Health Sex. 2014;16(4):426–39. doi: 10.1080/13691058.2014.887147 24697583

[57] Rodríguez-Camacho MF, Sanchís-Ramón MJ, Ortiz-Barreda G, La Parra-Casado D, Gil-González D. Menarche and reproductive health in Spanish Roma women from a reproductive justice perspective: a qualitative study. Reprod Health. 2024;21(1):17. doi: 10.1186/s12978-023-01726-5 38308316 PMC10837920

[58] Sommer M. Ideologies of sexuality, menstruation and risk: girls’ experiences of puberty and schooling in northern Tanzania. Cult Health Sex. 2009;11(4):383–98. doi: 10.1080/13691050902722372 19326264

[59] Santer M, Wyke S, Warner P. Women’s management of menstrual symptoms: findings from a postal survey and qualitative interviews. Soc Sci Med. 2008;66(2):276–88. doi: 10.1016/j.socscimed.2007.08.018 17920178

[60] Fehintola FO, Fehintola AO, Aremu AO, Idowu A, Ogunlaja OA, Ogunlaja IP. Assessment of knowledge, attitude and practice about menstruation and menstrual hygiene among secondary high school girls in Ogbomoso, Oyo state, Nigeria. Int J Reprod Contracept Obstet Gynecol. 2017;6(5):1726–32. doi: 10.18203/2320-1770.ijrcog20171932

[61] Diamond-Smith N, Onyango GO, Wawire S, Ayodo G. Knowledge of menstruation and fertility among adults in rural Western Kenya: Gaps and opportunities for support. PLoS One. 2020;15(3):e0229871. doi: 10.1371/journal.pone.0229871 32126117 PMC7053729

[62] Mimche H, Yongsi BN, Tamekeng M. Menstrual hygiene management: the experience of nomadic and sedentary populations in Niger. 2017.

[63] Mohamed Y, Durrant K, Huggett C, Davis J, Macintyre A, Menu S, et al. A qualitative exploration of menstruation-related restrictive practices in Fiji, Solomon Islands and Papua New Guinea. PLoS One. 2018;13(12):e0208224. doi: 10.1371/journal.pone.0208224 30507969 PMC6277107

[64] Iroegbu DI, Adamu VE, Ekhator J, Ojike AN, Oyama LE, Eze CC. Faith-based approach to improve menstrual hygiene management (MHM): challenges and successes [Internet]. 2018 [cited 2024 Oct 26]. Available from: https://repository.lboro.ac.uk/articles/conference_contribution/Faith-based_approach_to_improve_menstrual_hygiene_management_MHM_challenges_and_successes/9593084/1

[65] Maharaj T, Winkler IT. Transnational Engagements: Cultural and Religious Practices Related to Menstruation. In: Bobel C, Winkler IT, Fahs B, Hasson KA, Kissling EA, Roberts TA, editors. The Palgrave Handbook of Critical Menstruation Studies [Internet]. Singapore: Palgrave Macmillan; 2020 [cited 2024 Oct 26]. Available from: http://www.ncbi.nlm.nih.gov/books/NBK565655/33347200

[66] Rothbaum BO, Jackson J. Religious influence on menstrual attitudes and symptoms. Women Health. 1990;16(1):63–78. doi: 10.1300/J013v16n01_05 2309496

[67] Thapa S, Aro AR. “Menstruation means impurity”: multilevel interventions are needed to break the menstrual taboo in Nepal. BMC Womens Health. 2021;21(1):84. doi: 10.1186/s12905-021-01231-6 33639917 PMC7971149

[68] Benshaul-Tolonen A, Aguilar-Gomez S, Heller Batzer N, Cai R, Nyanza EC. Period teasing, stigma and knowledge: A survey of adolescent boys and girls in Northern Tanzania. PLoS One. 2020;15(10):e0239914. doi: 10.1371/journal.pone.0239914 33112868 PMC7592731

[69] Schmitt ML, Booth K, Sommer M. A policy for addressing menstrual equity in schools: A case study from New York City, U.S.A. Front Reprod Health. 2022;3:725805. doi: 10.3389/frph.2021.725805 36303999 PMC9580679

[70] Mason L, Nyothach E, Alexander K, Odhiambo FO, Eleveld A, Vulule J, et al. We keep it secret so no one should know – a qualitative study to explore young schoolgirls’ attitudes and experiences with menstruation in rural western Kenya. PLOS ONE. 2013;8(11):e79132. doi: 10.1371/journal.pone.0079132 PMC3828248 24244435

[71] Stoilova D, Cai R, Aguilar-Gomez S, Batzer NH, Nyanza EC, Benshaul-Tolonen A. PLOS Glob Public Health. 2022;2(3):e0000110. doi: 10.1371/journal.pgph.0000110 PMC10021794 36962274

[72] Tanton C, Nakuya K, Kansiime C, Hytti L, Torondel B, Francis SC, et al. Menstrual characteristics, menstrual anxiety and school attendance among adolescents in Uganda: a longitudinal study. BMC Womens Health. 2021;21(1):410. doi: 10.1186/s12905-021-01544-6 34895210 PMC8665501

[73] Miiro G, Rutakumwa R, Nakiyingi-Miiro J, Nakuya K, Musoke S, Namakula J, et al. Menstrual health and school absenteeism among adolescent girls in Uganda (MENISCUS): a feasibility study. BMC Womens Health. 2018;18(1):4. doi: 10.1186/s12905-017-0502-z 29298699 PMC5753466

[74] Girod C, Ellis A, Andes KL, Freeman MC, Caruso BA. Physical, Social, and Political Inequities Constraining Girls’ Menstrual Management at Schools in Informal Settlements of Nairobi, Kenya. J Urban Health. 2017;94(6):835–46. doi: 10.1007/s11524-017-0189-3 28875308 PMC5722726

[75] Buitrago-García T, Sawadogo NH, Souares A, Koulidiati JL, Sié A, Bärnighausen T, et al. Female-friendly toilets in schools in Burkina Faso: A mixed-methods study using photo-elicitation. J Glob Health. 2022;12:04057. doi: 10.7189/jogh.12.04057 36073661 PMC9454237

[76] Boosey R, Prestwich G, Deave T. Menstrual hygiene management amongst schoolgirls in the Rukungiri district of Uganda and the impact on their education: a cross-sectional study. Pan Afr Med J. 2014;19:253. doi: 10.11604/pamj.2014.19.253.5313 25852796 PMC4382073

[77] Ahmed MdS, Yunus FM, Hossain MdB, Sarker KK, Khan S. Association between Menstrual Hygiene Management and School Performance among the School-Going Girls in Rural Bangladesh. Adolescents. 2021;1(3):335–47. doi: 10.3390/adolescents1030025

[78] Grant M, Lloyd C, Mensch B. Menstruation and school absenteeism: evidence from rural Malawi. Comp Educ Rev. 2013;57(2):260–84. doi: 10.1086/669121 25580018 PMC4286891

[79] Shah V, Nabwera H, Sonko B, Bajo F, Faal F, Saidykhan M, et al. Effects of Menstrual Health and Hygiene on School Absenteeism and Drop-Out among Adolescent Girls in Rural Gambia. Int J Environ Res Public Health. 2022;19(6):3337. doi: 10.3390/ijerph19063337 35329020 PMC8954348

[80] Hennegan J. Understanding interventions to improve menstrual health in low and middle income countries: evidence and future directions. [Internet]. University of Oxford; 2017 [cited 2024 Oct 27]. Available from: https://ora.ox.ac.uk/objects/uuid:4afbd97f-52fe-459c-b82e-57045e598363

[81] Nalugya R, Tanton C, Hytti L, Kansiime C, Nakuya K, Namirembe P, et al. Assessing the effectiveness of a comprehensive menstrual health intervention program in Ugandan schools (MENISCUS): process evaluation of a pilot intervention study. Pilot Feasibility Stud. 2020;6:51. doi: 10.1186/s40814-020-00585-2 32346485 PMC7181508

[82] Poulose PA. More Than a Period: Creative Engagement and Storytelling to Transform the Menstrual Communication Landscape [Text] [Internet]. Emily Carr University of Art and Design. 2024 [cited 2024 Oct 27]. Available from: https://arcabc.ca/islandora/object/ecuad:18690doi:10.35010/ecuad:18690

[83] Warrington S, Coultas M, Das M, Nur E. “The door has opened”: moving forward with menstrual health programming in Bangladesh. Int J Hum Rights Healthc. 2021;14(4):296–310. doi: 10.1108/ijhrh-11-2020-0102

[84] Hiemstra R. Uses and benefits of journal writing. New Dir Adult Contin Educ. 2001;2001(90):19–26. doi: 10.1002/ace.17

[85] Kasprabowo T, Rahayu EY, Widyaningrum A. Tell me about your day: Portraying students’ reflective practice through diary writing. Lang Circ J Lang Lit. 2021;15(2):375–84. doi: 10.15294/lc.v15i2.29252

[86] Abramovich A, Huber HS. Personal reflective diaries with group-friendly criticism: empowering pre-service teachers. Teach Dev. 2024;28(1):122–40. doi: 10.1080/13664530.2023.2246929

[87] Broom AF, Kirby ER, Adams J, Refshauge KM. On Illegitimacy, Suffering and Recognition: A Diary Study of Women Living with Chronic Pain. Sociology. 2014;49(4):712–31. doi: 10.1177/0038038514551090

[88] Wilkinson S, Wilkinson C. Researching drinking “with” young people: a palette of methods. Drugs Alcohol Today. 2018;18(1):6–16. doi: 10.1108/dat-08-2017-0036

[89] Bartlett R. Modifying the diary interview method to research the lives of people with dementia. Qual Health Res. 2012;22(12):1717–26. doi: 10.1177/1049732312462240 23034779

[90] Day M, Thatcher J. I’m really embarrassed that you’re going to read this …: Reflections on using diaries in qualitative research. Qual Res Psychol. 2009;6(4):249–59. doi: 10.1080/14780880802070583

[91] Arndt H, Rose H. Capturing life as it is truly lived? Improving diary data in educational research. Int J Res Method Educ. 2023;46(2):175–86. doi: 10.1080/1743727x.2022.2094360

[92] Monrouxe LV. Solicited audio diaries in longitudinal narrative research: a view from inside. Qual Res. 2009;9(1):81–103. doi: 10.1177/1468794108098032

[93] Mupambireyi Z, Bernays S. Reflections on the Use of Audio Diaries to Access Young People’s Lived Experiences of HIV in Zimbabwe. Qual Health Res. 2019;29(5):680–92. doi: 10.1177/1049732318780684 29938607

[94] Kenten C. Narrating oneself: Reflections on the use of solicited diaries with diary interviews. Forum Qual Soc Res. 2010;11(2):2. doi: 10.17169/fqs-11.2.1314

[95] Zimmerman DH, Wieder DL. The diary: Diary-interview method. Urban Life. 1977;5(4):479–98. doi: 10.1177/089124167700500406

[96] Owen L. Stigma, sustainability, and capitals: A case study on the menstrual cup. Gend Work Organ. 2022;29(4):1095–112. doi: 10.1111/gwao.12808

[97] Hawkes G, Houghton J, Rowe G. Risk and worry in everyday life: Comparing diaries and interviews as tools in risk perception research. Health Risk Soc. 2009;11(3):209–30. doi: 10.1080/13698570902906439

[98] Oulo B, Sidle AA, Kintzi K, Mwangi M, Akello I. Understanding the barriers to girls’ school return: Girls’ voices from the frontline of the COVID-19 pandemic in East Africa. 2021.

[99] Betsu BD, Medhanyie AA, Gebrehiwet TG, Wall LL. Menstrual hygiene management interventions and their effects on schoolgirls’ menstrual hygiene experiences in low and middle countries: A systematic review. PLoS One. 2024;19(8):e0302523. doi: 10.1371/journal.pone.0302523 39172930 PMC11340951

