# Supplementary material for: Youth insights on menstrual health and hygiene in Burkina Faso; a comparison of written versus spoken qualitative text
Source: PLOS Glob Public Health. 2026 Sep 11;6(9):e0006804. doi: 10.1371/journal.pgph.0006804 (PMC13567792; doi:10.1371/journal.pgph.0006804)
Supplement: Supplementary file 3 [file pgph.0006804.s003.docx]

### **Inclusion & Exclusion Criteria**

Ethnicity, race, political orientation, religion, and class are not criteria for inclusion or exclusion in this study in order to allow for generalizability of the results within the Nouna region of Burkina Faso. In order to be able to reflect the ultimate goal of the later trial (which will evaluate the impact of the provision of MHM on school attendance), it was necessary to include girls based on the onset of menstruation (as young as 10). Given that we aim at determining a feasible and acceptable solution in MHM for women in the context of marginalized areas, we wish to be able to provide this to women from their menarche. Involving girls at younger ages in both menstrual hygiene education and safe menstrual practices through the provision of sanitary products is a way of addressing the difficulties and barriers related to this age group, which likely differ from those related to older women.

| **Inclusion criteria** | **Exclusion criteria** |
| --- | --- |
| Age of >10 or higher  Able to give informed consent if aged > 17  Willing and able to give informed assent to participate, and have a parent/guardian willing and able to give informed consent if aged <17 | Age <10  Incapacitated  Unable to give informed consent  Parent/guardian of participant refusing consent if aged <17  Participant refusing consent |
