# Supplementary material for: Youth insights on menstrual health and hygiene in Burkina Faso; a comparison of written versus spoken qualitative text
Source: PLOS Glob Public Health. 2026 Sep 11;6(9):e0006804. doi: 10.1371/journal.pgph.0006804 (PMC13567792; doi:10.1371/journal.pgph.0006804)
Supplement: Supplementary file 4 [file pgph.0006804.s004.docx]

**Interview Tool**

**Menstruation and Menstrual Hygiene Management: Experiences and Opportunities in rural Burkina Faso**

**In Depth Interview with School Girls**

1. Please tell me words that come to your mind when I say the word "menstruation". (*Write girl’s responses on a sheet of paper; probe on points that are especially interesting to us, so points on hygiene or sanitary products*)
2. Please describe any good things that you associate with having a period.
3. Please describe any bad things that you associate with having a period.
4. What are words you have used or heard when people talk about menstruation or girls who are menstruating?
5. What does it mean for a girl in Burkina Faso to start menstruating?
   a. How do people treat a girl before she menstruates compared to after she starts to menstruate?
6. Now I would like to talk about your own menstruation. You are welcome to tell me as much as you would like to tell. There is no right or wrong answer.
7. Did you learn about menstruation before having your first period?
   a. Who talked to you about it?
   b. How did the conversation go?
   c. Do you remember when you first got your period? Please walk me through that day.
      i. Did you tell anybody when you got your period for the first time?
      ii. How did you feel when your period came?
      iii. How would you describe your experience?”,
8. Nowadays, we see that girls around the world get information about their periods from all kinds of places. Where do you get information?
   a. Who do you talk about it with?
   b. If you had new doubts, who did you speak with? Why?
   c. What are your ideas to improve the menstrual experience?
   d. Why do you talk about it with this person and not with others?
   e. Do the teachers talk about it in school?
   f. Do you talk about it with your mom? Why or why not?
9. How was your most recent experience?
   a. Probe on preparation. (Please tell me whether you felt prepared? What would have made you feel more prepared?)
10. What do girls do if they get their periods in school?
    a. Who can they ask for help?
    b. What does the help consist of?
       i. Are there supplies at school?
       ii. What is privacy like?
       iii. Is there a place to dispose of sanitary products/device in school?
11. How do you manage your period at home?
    a. Where? Can you describe the place?
    b. What kind of things do you use?
    c. What is important to do?
12. How is the experience different from managing it at school?
13. Did you speak about this topic with friends at school?
14. Have you or girls you know ever missed school because of a period?
15. Now I'd like to talk about menstruation supplies. Which sanitary products do you know about? Which ones do you use?
    a. How long have you been using this product?
    b. Did you use another one before? Which one? Why did you change?
    c. What do you like/dislike about each product?
    d. Where do you keep your product(s)?
    e. Who provides these supplies to you?
    f. Is there another product you would like to use and if so, please tell me about that product.
       i. Why would you like to use that product?
16. What do you think would make it easier when you are menstruating?
    a. Which one of the products that we discussed do you think other girls in Nouna would like to use? (Would you like to try any of these products?)
    b. What are your concerns about them?
    c. What would be the best mechanism for distributing or creating sanitary products?
17. Is there anything else you would like to tell me about menstruation? About how girls manage their periods in Burkina Faso?
18. I'm happy to answer any questions you have.

**Thank you**
