# Supplementary material for: Youth insights on menstrual health and hygiene in Burkina Faso; a comparison of written versus spoken qualitative text
Source: PLOS Glob Public Health. 2026 Sep 11;6(9):e0006804. doi: 10.1371/journal.pgph.0006804 (PMC13567792; doi:10.1371/journal.pgph.0006804)
Supplement: Supplementary file 5 [file pgph.0006804.s005.docx]

**Interview Tool**

**Menstruation and Menstrual Hygiene Management: Experiences and Opportunities in rural Burkina Faso**

**In Depth Interviews with School Girls**

**Postdistribution**

1. The last time we met, Since the last time we met, how would you describe menstrual hygiene management now? Would you use the same words or do you have more words to describe it?
2. Now please tell me about the way you received the product and the feedback you got while you had the product.
   a. How did that conversation go?
   b. What more would you have liked to know?
   c. Where did you receive this product? Who gave you the product?
      i. Would you prefer to continue receiving the product this way, or would you rather receive the product in a different place?
      ii. Who might be a better person to receive the product from?
   d. Who guided you in terms of how to use this product?
   e. What would you make sure to explain to another friend who started using this product?
3. Please describe your experiences with (name the product used by the girl).*(Pay attention to signs that may indicate she might have not tried the product.)*
   a. What are some of the things that you did not like about the product?
   b. What are some of the things that you liked about this product?
   c. How was the experience changing it at school/home?
   d. If you could, would you want to use this product again, why or why not?
   e. Please compare this product to the approach you were using before.
   f. Did you use the product regularly during your last two periods? If not, what were the reasons that kept you from doing so?
   g. Now that you have tried this product, would you have liked to try any of the other products that we showed you last time? Which one? Why?
4. Now, I would like to talk about your experience with your diary. Let’s first start with some general questions. How did you feel about it?
   a. What did you like/dislike about it?
   b. What would you change in the diary?
   c. What would you add to it?
   d. Did you feel like you had enough space to express yourself?
   e. Did you find any difficulties in using it?

Probes: finding a place to write comfortably, privacy, other factors at home, features of the diary itself, keeping the habit of writing, others.

f. What would have made it easier for you?
g. How did you feel about writing in the diary?

5. Would you show me your diary so we can discuss a little bit more specifically? (*Check for questions that were left unanswered or answered very briefly in comparison to the others.*)
   a. Do you remember any questions that you liked more? Why?
   b. Can you show me some questions that you would have changed? What didn’t you like about them?
   c. What made you leave this question blank (*or write less than for the others*)?
   d. Can you think of any answers for them now? Is there anything you want to add about these questions?
   e. What parts of the diary did you enjoy more?
   f. Was it easier to write freely or do you prefer the part with questions?
   g. Can you think of any questions you would have added? What are some questions that you think other girls filling in the diary would have like to talk about?
6. What about your general experience since the beginning, since our first group interview?
   a. What are your general thoughts?
   b. Was it like you expected it?
   c. Do you remember anything you didn’t like?
   d. Was there anything you liked?
   e. What was your favorite part? Why?
   f. Is there anything else that we haven’t discussed till now that you would have changed?
7. As you know, all steps in this project are meant to help us understand how we can better develop a program to help girls like you. This is why your opinion is most important and why now I would like to imagine with you how that ideal program would look like from the answers you have given. I will go ahead and describe it from the notes I have but I need you stop me any time that I say something you don’t agree with or that you think of something that you would like to add.

*(Please make this summary from her own answers. Envision the program WITH HER, encourage her repeatedly to intervene and add to your description. Describe the program IN DETAIL. Here you can find an example.)*

In this ideal program, girls from schools would be chosen … *(Way of selection of partcipants)*

They would also be able to… *(Specifics about the selection of the product or general changes regarding distribution and the products that the girl might have proposed during the interview)*

They have … *(time to think about the products before choosing).*

The products are given out to all the girls… *(Individually? All girls together? All girls using the same product together? With or without mothers present?)*

The best/ideal place for distribution of the products would be…

The person distributing the products would be…

This person should explain… *(things that this person should explain to then when giving out the product)*

If a girl has questions after the distribution of the product, they can approach … (*person they can contact in case any more doubts arise)*

Other people that should learn about menstrual hygiene management and the products are…

The diary should be… (*describe the diary, characteristics and changes she has proposed)*

Additional things that are important for this ideal program are… *(Other changes or important points she has talked about during the interview)*

Is this everything? Can you think of anything else you would like to add to this description?

8. Is there anything else you would like to tell me about using this product? About how girls manage their periods in Burkina Faso?
9. I'm happy to answer any questions you have.

**Thank you**
